# Psychotropic Medication Prescriptions and Large California Wildfires

**DOI:** 10.1001/jamanetworkopen.2023.56466

**Published:** 2024-02-26

**Authors:** Zachary S. Wettstein, Ambarish Vaidyanathan

**Affiliations:** 1 Climate and Health Program, National Center for Environmental Health, Centers for Disease Control and Prevention, Atlanta, Georgia; 2 University of Washington School of Medicine, Department of Emergency Medicine, Seattle

## Abstract

**Importance:**

Wildfires, intensified by climate change, have known effects on physical health but their effects on mental health are less well characterized. It has been hypothesized that the residential proximity to a large wildfire can exacerbate underlying mental health conditions as evidenced by increased prescriptions of psychotropic medications.

**Objective:**

To evaluate the association between the occurrence of large wildfires and the prescription rates of psychotropic medications immediately following the start of the fire.

**Design, Setting, and Participants:**

This cohortstudy used an interrupted time-series analysis to compare psychotropic medication prescriptions in the 6 weeks before and after each of 25 wildfires. The setting was California counties within metropolitan statistical areas (MSAs) experiencing large wildfires from 2011 through 2018. Participants included individuals residing in California MSAs with prescriptions of psychotropic medications recorded in the Merative MarketScan Research Database (MarketScan) during the study period. Statistical analysis was performed for these 25 large wildfires occurring between September 2011 and November 2018.

**Exposure:**

Residential proximity to large wildfires that burned more than 25 000 acres occurring in a California county within an MSA.

**Main Outcomes and Measures:**

Prescriptions of psychotropic medications, including antidepressants, antipsychotics, anxiolytics, hypnotics, and mood-stabilizers, with statins as a negative control outcome.

**Results:**

For the study period, prescription data and patient-level attributes were extracted for 7 115 690 unique individuals (annual mean [range]: 889 461 [455 705-1 426 928] individuals) enrolled in MarketScan and residing in fire-affected MSAs. This study found a statistically significant increase in prescriptions of antidepressants (rate ratio [RR], 1.04 [95% CI, 1.01-1.07]), anxiolytics (RR, 1.05 [95% CI, 1.02-1.09]), and mood-stabilizing medications (RR, 1.06 [95% CI, 1.01-1.13]) in the fire period compared with the prefire baseline. However, the prescriptions of antipsychotics, hypnotics, and the negative control outcome, statins, showed no significant association.

**Conclusions and Relevance:**

In this cohort study of large California wildfires, the occurrence of wildfire was associated with increased mental health burden as reflected in increased prescription rates of certain psychotropic medications. The findings underscore the need for further scientific examination into the mental health effects of wildfires and the allocation of mental health resources in disaster responses. California experienced a substantial burden of wildfires from 2011 to 2018, and as wildfires become more intense and frequent in the context of anthropogenic climate change, it is increasingly important to understand and address their mental health effects.

## Key Points

**Question:**

Is residential proximity to large wildfires associated with increased prescriptions of psychotropic medications, indicating greater mental health burden?

**Findings:**

This cohort study of more than 7 million individuals living in areas affected by 25 large California wildfires (2011-2018) found a statistically significant increase in prescriptions of antidepressants, anxiolytics, and mood-stabilizing medications during the fire period compared with the prefire period, but not for antipsychotics, hypnotics, or the negative control outcome, statins.

**Meaning:**

This study’s results suggest that large wildfires may exacerbate certain mental health conditions, necessitating a comprehensive public health approach that ensures access to a wide variety of services, including those that improve mental health resilience before, during, and after disasters.

## Teaser

This cohort study investigates whether there is an association between large wildfires and the prescription rates of psychotropic medications immediately following the start of the fire.

## Introduction

In recent years, states in the western United States have experienced destructive wildfires with substantial societal effects.^1,2^ While wildfires can occur naturally and many ecosystems benefit from periodic fires, climate change has been a sizable contributor to the increased frequency, intensity, and duration of large wildfires.^1^ Consequently, wildfire-related damages to property and disruptions to people’s life have become more frequent in the last few decades, especially in California.^3,4,5,6^

The public health burden due to wildfires can be substantial with negative health consequences resulting from direct fire-related damage, air pollution, and severe disruptions to ecosystems. While the physical health effects of wildfires have been extensively studied, their effect on mental health is poorly understood despite a vast burden expected.^3,4,7,8,9^ Prior studies assessing mental health outcomes associated with disasters have identified increases in diagnoses and medication prescriptions for anxiety, depression, posttraumatic stress disorder (PTSD), and sleep disturbances associated with earthquakes, hurricanes, and floods.^10,11^ Few studies have explored the implications of wildfires on mental health, which remain challenging to study due to a range of factors including stigma, underdiagnosis, and difficulty in measuring mental health burden.^11,12^ Furthermore, published studies have identified associations between wildfire and impaired mental health including anxiety, depression, and PTSD, but these studies have relied primarily on qualitative methods or focused on a particular wildfire or season; and, to our knowledge, none have used prescriptions of psychotropic medications as a proxy for mental health burden nor evaluated across wildfire seasons.^3,4,7,8,9,13^

We hypothesized that the presence of large wildfires in California, especially near population centers, would be associated with exacerbations of underlying mental health conditions. Toward this end, our objective for this study was to analyze associations between large wildfires in California and prescription rates of various psychotropic medication categories as a proxy indicator of public mental health burden.

## Methods

### Setting and Data Sources

We conducted a cohort study analyzing psychotropic medication prescriptions in California around the times of 25 of the largest wildfires that occurred in California from 2011 through 2018. Our fire selection criteria were based on acreage burned (>25 000 acres) and occurrence within a county that was part of a metropolitan statistical area (MSA) to account for proximity to the fires. We identified fire size using the California Department of Forestry and Fire Protection (CALFIRE) Redbook (eTable 1 in Supplement 1). We obtained information on outpatient drug claims from the Merative MarketScan Research Database (MarketScan; commercial claims and encounters data, 2011-2018). All health data used in this study were deidentified, so the study did not qualify as human research according to the policies of the US Centers for Disease Control and Prevention (CDC); therefore, institutional review board approval and informed consent were not required.

### Outcome Measures

We extracted daily medication prescriptions from MarketScan with information on the MSA that each patient resided in along with patient-level attributes, such as age and sex. In addition, we categorized them into various psychotropic medication groups, according to Micromedex, with statins included as a control category (eTable 2 in Supplement 1). Specifically, we created 7 daily time series at the MSA level: (1) all psychotropic medications, (2) antidepressants, (3) antipsychotics, (4) anxiolytics, (5) hypnotics, (6) mood stabilizers, and (7) statins. Additional analyses were stratified by age group (18-44 years and 45-64 years) and sex (male or female) for all outcomes. We identified the onset date of a wildfire episode as the first date of posting on Twitter by an official local, state, or federal wildfire agency. Tweets have been traditionally used by wildfire agencies to provide real-time updates on containment efforts and alert the public of fire-related hazards. Based on the posting date, we restricted the duration of each wildfire-specific study period to 6 weeks after the start of the fire (fire period) and 6 weeks before fire initiation (prefire period). For each series, we extracted the total number of persons enrolled in MarketScan during the fire and prefire period. Our analysis did not consider a stratified analysis by race or ethnicity as that information was not available from MarketScan. This study followed the Strengthening the Reporting of Observational Studies in Epidemiology (STROBE) reporting guideline.

### Statistical Analysis

We used an interrupted time-series (ITS) regression framework to explore the association of wildfire episodes and the prescription rates of psychotropic medications.^14,15,16,17^ For our analysis, we used the first Twitter posting date for each wildfire as the interruption point. Based on this interruption point, we performed an ITS analysis comparing the 6 weeks after the start of the fire with the 6 weeks before fire initiation. We relied on recently published ITS studies to account for variables representing time, including a variable to denote time since the start of each wildfire-specific study period that was centered at the interruption point and a binary indicator based on the interruption point, denoting the mean change in daily prescription medication between prefire and fire periods. Specifically, we fitted Poisson generalized linear models, that accounted for overdispersion.^18^ We regressed daily counts of various psychotropic medication categories against the binary variable denoting mean change in daily prescription medications, while controlling for time trends, seasonality, and factors that influence baseline prescription rates for each MSA where a major wildfire episode occurred. Of note, using a binary indicator, we controlled for the cooccurrence of any extreme weather alerts and disaster declarations for other hazards (eg, cold, flood, and heat alerts), reported in the National Oceanic and Atmospheric Administration (NOAA) storm event database, either during the fire or prefire period.^19^ Other variables included a variable for the combination of wildfire name and location, an interaction between month and year, an indicator to identify weekends, and an interaction term between the time variable centered at the interruption point and the variable denoting name and location of the wildfire. We used the logarithm of persons enrolled in MarketScan during the fire and prefire period as an offset variable to adjust for potential fluctuations in enrollee population over time. The models estimated mean daily change in prescription rates between fire and prefire periods as rate ratios (RRs) with 95% CIs and were implemented using generalized estimating equations with a first-order autoregressive correlation structure to allow for correlation within MSA. A 2-sided α of .05 was used for statistical significance. We performed data analysis using SAS version 9.4 (SAS Institute) and ArcGIS version 10.8 (Environmental Systems Research Institute). Statistical analysis was performed for these 25 large wildfires occurring between September 2011 and November 2018.

We also conducted a series of sensitivity analyses to examine the robustness of results. First, we identified an alternative interruption point based on the wildfire onset date reported in the CALFIRE Redbook. In addition, we also explored the sensitivity associated with the length of prefire and fire period by varying the duration to 4, 8, or 12 weeks. Lastly, we executed models by controlling for binary indicators for specific extreme weather events or disasters, and continuous measures of daily temperature and air pollution. We obtained daily mean air temperature in degrees Fahrenheit (°F) from NOAA National Center for Environmental Information and used estimates of daily 24-hour mean fine particulate matterin micrograms per cubic meter and daily maximum 8-hour mean ozone concentrations in parts per billion from a bayesian space-time downscaler fusion model.^20,21,22^

## Results

This study included 25 fires spread across 14 MSAs (Figure). Between January 1, 2011, and December 31, 2018, we extracted patient-level attributes for 7 115 690 unique individuals (annual mean [range]: 889 461 [455 705-1 426 928] individuals) enrolled in MarketScan and residing in fire-affected MSAs. The mean (range) percentage of enrollees who had at least 1 outpatient drug claim was 22% (21%-23%) for psychotropic medications and 12% (11%-13%) for statin medications. Overall, medication use patterns were different between the prefire and the fire period; of note, 251 978 psychotropic prescriptions were used in the prefire period vs 255 319 in the fire period, and 97 463 statin prescriptions were used in the prefire period vs 97 764 in the fire period. Age-specific and sex-specific distributions of psychotropic and statin medications are provided in Table 1.

**Figure.**
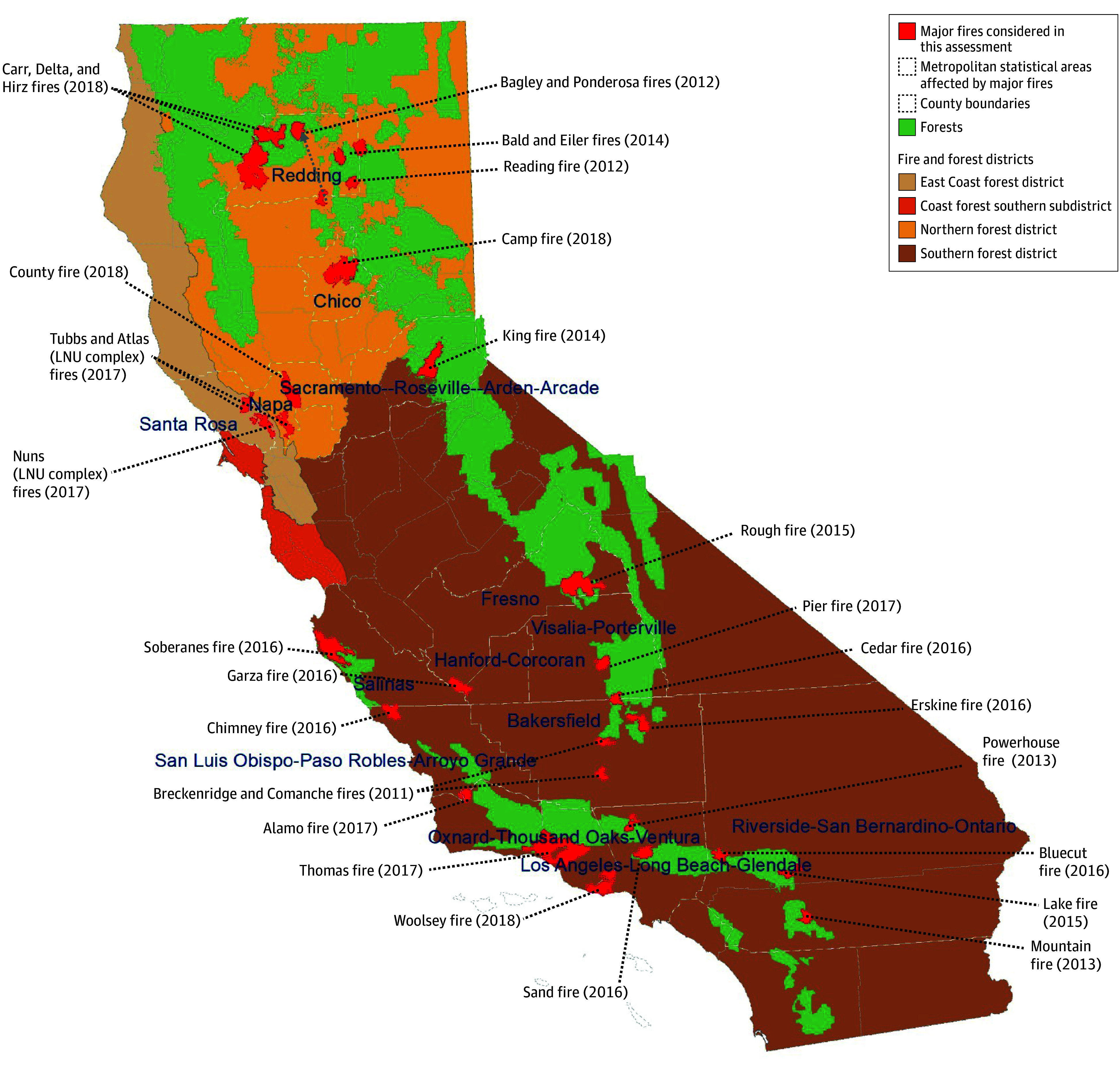
Map of California Fire and Forest Districts, Major Fires Considered in the Analysis, and Metropolitan Statistical Areas Affected by Major Fires From 2011 to 2018 LNU indicates Sonoma-Lake-Napa Unit.

**Table 1.** Summary of Psychotropic and Stain Medication Fills Stratified by Sex, Age Group, and Prefire and Fire Periods

Medication class	Sex	Medication fills, No.					
		All ages		18-44 y		45-64 y	
		Prefire period	Fire period	Prefire period	Fire period	Prefire period	Fire period
All psychotropic medications	All sexes	251 978	255 319	92 595	94 038	148 143	149 944
	Female	164 564	167 239	59 816	61 236	99 135	100 303
	Male	87 414	88 080	32 779	32 802	49 008	49 641
Antidepressant medications	All sexes	133 771	135 681	51 249	52 085	76 398	77 366
	Female	91 747	93 127	34 741	35 466	53 616	54 217
	Male	42 024	42 554	16 508	16 619	22 782	23 149
Antipsychotic medications	All sexes	14 276	14 493	6247	6448	5750	5780
	Female	7567	7820	3050	3278	3704	3724
	Male	6709	6673	3197	3170	2046	2056
Anxiolytic medications	All sexes	76 222	77 585	25 385	25 734	49 235	50 234
	Female	49 392	50 543	16 374	16 750	32 199	32 964
	Male	26 830	27 042	9011	8984	17 036	17 270
Hypnotic medications	All sexes	25 198	25 286	6713	6702	18 440	18 544
	Female	15 007	15 074	4033	4055	10 954	10 998
	Male	10 191	10 212	2680	2647	7486	7546
Mood-stabilizer medications	All sexes	12 157	12 332	5904	6130	4830	4753
	Female	7241	7483	3492	3726	3056	3014
	Male	4916	4849	2412	2404	1774	1739
Statin medications (negative control outcomes)	All sexes	97 463	97 764	10 234	10 162	87 167	87 547
	Female	38 016	38 199	2739	2776	35 245	35 398
	Male	59 447	59 565	7495	7386	51 922	52 149

In the primary analysis using fire start according to first posting on Twitter, we observed statistically significant RRs, indicating an increase in mean daily medication prescriptions for the fire period, for antidepressant medications (1.04 [95% CI, 1.01-1.07]), anxiolytic medications (1.05 [95% CI, 1.02-1.09]), mood-stabilizing medications (1.06 [95% CI, 1.01-1.13]), and all psychotropic medications (1.04 [95% CI, 1.01-1.07]) (Table 2). After age group–based stratification, these significant findings persisted for all medication categories, but category-specific associations were found only for older adults (aged 45-64 years) (Table 2). The effect sizes based on sex-stratification were comparable; however, RR for mood-stabilizing medications was only statistically significant for female enrollees (1.09 [95% CI, 1.02-1.17]). For antipsychotic medications (eg, RR for all ages and sexes, 1.01 [95% CI, 0.96-1.06]) and hypnotic medications (eg, RR for all ages and sexes, 1.04 [95% CI, 0.99-1.08]), mean RRs were greater than unity but the risk estimates were not statistically significant. Daily prescriptions for the control category of statins showed no statistically significant association during the period with wildfires (RR, 1.03 [95% CI, 1.00-1.06]) or across any stratifications considered in this analysis.

**Table 2.** Mean Daily Change in Psychotropic Medication Prescriptions in 6 Weeks After Wildfire Start Date Compared With 6-Week Period Preceding Fires, Across 25 Large California Wildfires

Medication type	Mean rate ratio (95% CI)				
	All ages and sexes	All sexes and ages 18-44 y	All sexes and ages 45-64 y	All ages and female	All ages and male
Psychotropic medications					
All psychotropic medications	1.04 (1.01-1.07)	1.03 (1.00-1.06)	1.05 (1.02-1.08)	1.05 (1.02-1.08)	1.04 (1.00-1.07)
Antidepressant medications	1.04 (1.01-1.07)	1.03 (0.99-1.06)	1.04 (1.01-1.07)	1.04 (1.01-1.07)	1.03 (0.99-1.07)
Antipsychotic medications	1.01 (0.96-1.06)	1.01 (0.94-1.09)	1.00 (0.93-1.08)	1.04 (0.97-1.11)	1.02 (0.95-1.10)
Anxiolytic medications	1.05 (1.02-1.09)	1.03 (1.00-1.08)	1.06 (1.03-1.10)	1.06 (1.02-1.09)	1.05 (1.00-1.09)
Hypnotic medications	1.04 (0.99-1.08)	1.07 (1.00-1.15)	1.03 (0.98-1.08)	1.02 (0.97-1.08)	1.05 (0.99-1.11)
Mood-stabilizer medications	1.06 (1.01-1.13)	1.08 (1.00-1.17)	1.02 (0.94-1.11)	1.09 (1.02-1.17)	1.03 (0.95-1.12)
Negative control outcome					
Statin medications	1.03 (1.00-1.06)	1.02 (0.96-1.09)	1.03 (1.00-1.06)	1.03 (0.99-1.07)	1.02 (0.99-1.05)

In the sensitivity analysis, when the fire start was based upon the CALFIRE Redbook, the results were consistent with Twitter-based wildfire onset date across all medication categories except for mood-stabilizer medications, and the hypnotic medications category was statistically significant for male enrollees (eTable 3 in Supplement 1). In addition, we observed that the length of the prefire and fire period did influence the risk estimates for all medication categories. Optimal length of the prefire and fire period seemed to be 6 weeks as there was a reduction in effect sizes beyond this duration and for certain medication categories, longer durations resulted in nonsignificant results. Of note, the addition of continuous measures of air pollution and temperature parameters and/or the inclusion of binary indicators for specific extreme weather events or disasters in the Poisson generalized linear models did not significantly affect associations between medication prescriptions and the binary indicator based on the interruption point, representing mean daily change (eFigure 1 and eFigure 2 in Supplement 1).

## Discussion

In this cohort study, psychotropic medication prescriptions increased in the fire period compared with the prefire baseline, including for antidepressant medications, anxiolytic medications, and mood-stabilizer medication, but not for antipsychotic medications, hypnotic medications, or statin medications, the nonpsychotropic control. Furthermore, by controlling for other severe weather events and disaster declarations, this analysis established a robust baseline and limited the potential confounding of other environmental hazards.

Possible mechanisms for health effects of wildfires have been previously discussed, and likely represent a range of factors resulting from the physical, psychological, and economic tolls imposed from wildfires.^3,4,7,10,13^ Mental health burdens likely result from physical health effects including trauma and cardiorespiratory conditions; distress reactions including sleep disruption and decreased sense of safety, particularly among those who face displacement, evacuation, and property loss; and exacerbations of underlying psychiatric disorders such as PTSD, anxiety, depression, and complex grief.^10^ While other analyses of wildfire health effects typically focus on air quality, there are numerous other hazards from wildfires ranging from direct flame exposure, ash and smoke constituents, water quality degradation, and property destruction. By focusing on residential proximity to the wildfires, this analysis considered the exposure to these numerous hazards as well as the community stress, anxiety, and social medial exposure to images and discussions of the wildfire that would be simultaneously present, which was hypothesized to be among the primary mechanisms of the resulting mental health effects.^23^

The sensitivity analyses further supported the robustness of the findings in this study. Controlling for temperature, precipitation, and air pollution demonstrated some degree of confounding but with a persistent and similar distribution of the results, suggesting that the effect of these fires on mental health burden may be due to a separate mechanism, such as community stress and social media exposure to wildfire-related content. Stratification of the analysis by age was statistically significant in all psychotropic medications regardless of age group, although with older adults demonstrating a greater magnitude of the effect, with statistically significant associations persisting for specific medication classes, including antidepressants and anxiolytics. This may have been due to an increased mental health burden among older adults associated with the wildfires; however, the greater sample size of psychotropic prescriptions among older adults may have contributed to this statistically significant finding as well. Interestingly, the sex-stratified results demonstrated a higher baseline count of psychotropic prescriptions among female enrollees, which may indicate a higher engagement in mental health care or a higher burden of mental health conditions among female individuals.^24,25,26^ Notably, the increase in psychotropic prescriptions in the postfire period were disproportionately higher among female enrollees than male enrollees, suggesting a higher engagement in mental health care and psychotropic medication use, and potentially greater mental health burden among female enrollees.

### Limitations

There were limitations to this study. The ITS framework can misrepresent the step change associated with the fire period, especially when other California-specific unplanned major events that occur simultaneously contribute to changes in prescription rates.^27^ In order to account for such uncertainties, we controlled for other disasters, restricted our fire and prefire periods to a reasonably short duration, and conducted a series of sensitivity analyses to ascertain the influence of several continuous environmental parameters. These additional steps helped verify the robustness of our results and reduced the possibility of misattributing the associations between wildfires and prescription rates. In addition, although this study did not evaluate whether these were new prescriptions or refills of prior medication, prior studies have found disasters associated with incident mental health diagnoses and prescriptions.^10^ As these prescription records were sourced from commercial claims data, they likely underestimate the burden for other disproportionately affected groups, such as people who are uninsured or experiencing unstable housing, and those enrolled in Medicaid or Medicare. Rural communities also face a disproportionate burden of wildfire exposure and concomitant lack of mental health resources, which may not be captured in these psychotropic medication prescriptions if conditions go undiagnosed and untreated, and they are likely underrepresented in this data set focused on MSAs.^3^ Additionally, individuals with limited or no access to mental health services are not likely included in this commercial claims database. Therefore, these findings may vastly underestimate the true risk of mental health effects among those not actively engaged in mental health care, who may be at greater risk without a preexisting structure for support and treatment.^11^

## Conclusions

In this cohort study of large California wildfires from 2001 to 2018, the analysis demonstrated a significant association between psychotropic medication fills and wildfires consistent with previous literature describing mental health risk associated with wildfires, but on a broader spatial and timescale using administrative prescription data.^3,4,7,8,9,13,28^ These findings warrant further examination of mental health effects related to wildfires, including exploration of exposure pathways and mechanisms for mental health effects, incident diagnoses compared with exacerbation of preexisting mental health conditions, and identification of mental health needs in communities and populations disproportionately affected by wildfires. Further investigation on mental health prescription medications is needed, particularly focusing on populations with limited or no access to mental health services, so that appropriate public health measures can be implemented with an emphasis on addressing medication needs of affected populations. In addition, the importance of clinical and public health efforts that shed light on negative mental health consequences is underscored by climate change and its role in increasing the frequency, intensity, and extent of wildfires and other environmental hazards, potentially escalating mental health burdens worldwide. Lastly, while awaiting further scientific exploration, ensuring access to mental health services and supporting programs that promote mental health resilience before, during, and after wildfires are integral to mitigating the mental health effects associated with catastrophic fires.^29,30^

## Notes

Supplement 1. **eTable 1.** Fires Greater than 25,000 Acres in Counties with Metropolitan Statistical Areas in California, According to the CALFIRE Redbook: 2011 Through 2018 **eTable 2.** Psychotropic and Statin Medication Classification According to Micromedex Red Book **eTable 3.** Mean Daily Change in Psychotropic Medication Prescriptions in 6 Weeks After Wildfire Start Date Posted on CALFIRE Redbook, Compared to 6 Week Period Preceding Fires, Across 25 Large California Wildfires in California MSAs: 2011-2018 **eFigure 1.** Comparison of Mean Daily Change in Psychotropic Medication Prescriptions by Different Pre-Fire and Fire Periods (Start Date Posted on Twitter), Across 25 Large California Wildfires in California MSAs: 2011-2018 **eFigure 2.** Comparison of Mean Daily Change in Psychotropic Medication Prescriptions After Controlling for Extreme Weather Events or Disasters, Meteorological Variables, and Air Pollution Parameters (Start Date Posted on Twitter), Across 25 Large California Wildfires—Results From Sensitivity Analysis

Supplement 2.
Data Sharing Statement
