# Supplementary material for: Psychotropic Medication Prescriptions and Large California Wildfires
Source: JAMA Netw Open. 2024 Feb 26;7(2):e2356466. doi: 10.1001/jamanetworkopen.2023.56466 (PMC10897744; doi:10.1001/jamanetworkopen.2023.56466)
Supplement: Supplement 1. — eTable 1. Fires Greater than 25,000 Acres in Counties with Metropolitan Statistical Areas in California, According to the CALFIRE Redbook: 2011 Through 2018 eTable 2. Psychotropic and Statin Medication Classification According to Micromedex Red Book eTable 3. Mean Daily Change in Psychotropic Medication Prescriptions in 6 Weeks After Wildfire Start Date Posted on CALFIRE Redbook, Compared to 6 Week Period Preceding Fires, Across 25 Large California Wildfires in California MSAs: 2011-2018 eFigure 1. Comparison of Mean Daily Change in Psychotropic Medication Prescriptions by Different Pre-Fire and Fire Periods (Start Date Posted on Twitter), Across 25 Large California Wildfires in California MSAs: 2011-2018 eFigure 2. Comparison of Mean Daily Change in Psychotropic Medication Prescriptions After Controlling for Extreme Weather Events or Disasters, Meteorological Variables, and Air Pollution Parameters (Start Date Posted on Twitter), Across 25 Large California Wildfires—Results From Sensitivity Analysis [file jamanetwopen-e2356466-s001.pdf]

Supplemental Online Content

Wettstein ZS, Vaidyanathan A. Psychotropic medication prescriptions and large California wildfires. *JAMA Netw Open.* 2024;7(2):e2356466. doi:10.1001/jamanetworkopen.2023.56466

eTable 1. Fires Greater than 25,000 Acres in Counties with Metropolitan Statistical Areas in California, According to the CALFIRE Redbook : 2011 Through 2018

eTable 2. Psychotropic and Statin Medication Classification According to Micromedex Red Book

eTable 3. Mean Daily Change in Psychotropic Medication Prescriptions in 6 Weeks After Wildfire Start Date Posted on CALFIRE Redbook, Compared to 6 Week Period Preceding Fires, Across 25 Large California Wildfires in California MSAs: 2011-2018

eFigure 1. Comparison of Mean Daily Change in Psychotropic Medication Prescriptions by Different Pre-Fire and Fire Periods (Start Date Posted on Twitter), Across 25 Large California Wildfires in California MSAs: 2011-2018

eFigure 2. Comparison of Mean Daily Change in Psychotropic Medication Prescriptions After Controlling for Extreme Weather Events or Disasters, Meteorological Variables, and Air Pollution Parameters (Start Date Posted on Twitter), Across 25 Large California Wildfires—Results From Sensitivity Analysis

This supplemental material has been provided by the authors to give readers additional information about their work.

© 2024 Wettstein ZS et al. *JAMA Network Open.*

eTable1. Fires Greater than 25,000 Acres in Counties with Metropolitan Statistical areas in California, According to the CALFIRE Redbook[1]: 2011 through 2018

**Wildfire Name**	**Metropolitan Statistical Area (MSA)**	**CALFIRE Redbook Start Date**	**Twitter-based Start Date**	**Acres Burned**	**Average Daily Population Enrolled in Merative MarketScan® Research Database**	
					**Pre-Fire Period**	**Fire Period**
Breckenridge & Comanche Complex	Bakersfield	9/10/2011	9/11/2011	54,551	121,247	121,229
Reading	Redding	7/23/2012	7/23/2012	28,079	31,137	31,147
Bagley & Ponderosa	Redding	8/18/2012	8/18/2012	73,687	31,144	31,143
Powerhouse	Los Angeles-Long Beach-Anaheim	5/30/2013	5/30/2013	30,274	993,740	993,803
Mountain	Riverside-San Bernardino-Ontario	7/15/2013	7/15/2013	27,531	366,586	366,566
Bald & Eiler	Redding	7/30/2014	8/3/2014	72,152	29,618	29,609
King	Sacramento-Roseville-Folsom	9/13/2014	9/14/2014	97,717	191,218	191,191
Lake	Riverside-San Bernardino-Ontario	6/17/2015	6/19/2015	31,359	99,272	99,284
Rough	Fresno	7/31/2015	8/11/2015	151,623	24,436	24,419
Erskine	Bakersfield	6/23/2016	6/23/2016	48,019	12,865	12,857
Sand	Los Angeles-Long Beach-Anaheim	7/22/2016	7/22/2016	41,383	374,153	374,082
Soberanes	Salinas	7/22/2016	7/22/2016	132,127	10,771	10,768
Chimney	San Luis Obispo-Paso Robles	8/13/2016	8/13/2016	46,235	4,030	4,031
Cedar	Bakersfield	8/16/2016	8/16/2016	29,322	12,874	12,910
Bluecut	Riverside-San Bernardino-Ontario	8/16/2016	8/18/2016	36,274	102,868	102,907
Alamo	San Luis Obispo-Paso Robles	7/6/2017	7/6/2017	28,687	4,055	4,057
Garza	Hanford-Corcoran	7/9/2017	7/14/2017	48,660	2,411	2,415
Pier	Visalia	8/29/2017	8/31/2017	36,556	7,187	7,181
Atlas & Tubbs	Napa	10/9/2017	10/9/2017	88,431	2,033	2,040
Nuns	Santa Rosa-Petaluma	10/9/2017	10/9/2017	44,573	11,504	11,505
Thomas	Oxnard-Thousand Oaks-Ventura, Santa Maria-Santa Barbara	12/4/2017	12/4/2017	281,893	37,837	38,608
County	Sacramento-Roseville-Folsom	6/30/2018	6/30/2018	90,000	87,779	87,828
Carr, Hirz, Delta	Redding	7/23/2018	7/23/2018	339,013	4,369	4,381
Camp	Chico	11/8/2018	11/8/2018	153,336	3,874	3,864
Woolsey	Oxnard-Thousand Oaks-Ventura	11/8/2018	11/8/2018	96,949	40,510	40,533

[1] https://www.fire.ca.gov/our-impact/statistics

© 2024 Wettstein ZS et al. *JAMA Network Open.*

eTable 2. Psychotropic and Statin Medication Classification According to Micromedex Red Book.[2]

Medication Class	Medication Subtype	Medications
Antidepressants	Selective serotonin reuptake inhibitors	Chlorprothixene, citalopram, escitalopram, fluoxetine, fluvoxamine, paroxetine, sertraline
	Serotonin noradrenaline reuptake inhibitors	Desvenlafaxine, duloxetine, levomilnacipran, venlafaxine
	Atypical antidepressants	Buproprion, mirtazapine
	Serotonin modulators	Nefazodone, trazodone, vilazodone, vortioxetine
	Tricyclic antidepressants	Amitriptyline, amoxapine, clomipramine, desipramine, doxepin, imipramine, maprotiline, nortriptyline, protriptyline, trimipramine
	Monoamine oxidase inhibitors	Isocarboxazid, phenelzine, selegiline, tranylcypromine
Antipsychotics	Typical antipsychotics	Acetophenazine, chlorpromazine, droperidol, fluphenazine, haloperidol, loxapine, mesoridazine, molindone, perphenazine, pimozide, piperacetazine, prochlorperazine, promazine, thioridazine, thiothixene, trifluoperazine, triflupromazine
	Atypical antipsychotics	Aripiprazole, asinapine, brexpiprazole, cariprazine, clozapine, iloperidone, lurasidone, olanzapine, paliperidone, pimavanserin, quetiapine, risperidone, ziprasidone
Anxiolytics	Benzodiazepines	Alprazolam, chlordiazepoxide, clorazepate, diazepam, estazolam, flurazepam, halazepam, lorazepam, midazolam, oxazepam, prazepam, quazepam, temazepam, triazolam
	Buspirone	Buspirone
	Gabapentin	Gabapentin
	Hydroxyzine	Hydroxyzine
Mood stabilizers	Lithium	Lithium
	Anti-epileptics	Carbamazepine, lamotrigine, oxcarbazepine, valproic acid
Hypnotics	Melatonin receptor agonists	Melatonin, ramelteon, tasimelteon
	Orexin antagonists	Suvorexant
	Z-drugs	Eszopiclone, zaleplon, zolpidem
Control (statins)	HMG CoA reductase inhibitors	Atorvastatin, cerivastatin, fluvastatin, lovastatin, pitavastatin, pravastatin, rosuvastatin, simvastatin

[2] https://www.nlm.nih.gov/research/umls/rxnorm/sourcereleasedocs/mmx.html

© 2024 Wettstein ZS et al. *JAMA Network Open.*

eTable 3. Mean Daily Change in Psychotropic Medication Prescriptions in 6 Weeks After Wildfire Start Date Posted on CALFIRE Redbook, Compared to 6 Week Period Preceding Fires, Across 25 Large California Wildfires in California MSAs: 2011-2018.

**Medication Type**	**Mean Rate Ratio (95%CI) for overall and stratified analyses by age group and sex**				
	All ages and sexes	***All sexes and ages, 18-44***	***All sexes and ages, 45-64***	***All ages and female***	***All ages and male***
Psychotropic Medications					
All Psychotropic Medications	**1.04 (1.01, 1.07)**	1.03 (0.99, 1.06)	**1.05 (1.02, 1.08)**	**1.04 (1.01, 1.07)**	1.03 (1.00, 1.07)
Antidepressant Medications	**1.03 (1.01, 1.06)**	1.02 (0.99, 1.06)	**1.04 (1.01, 1.07)**	**1.04 (1.01, 1.07)**	1.03 (0.99, 1.07)
Antipsychotic Medications	1.03 (0.97, 1.08)	1.03 (0.95, 1.11)	1.02 (0.94, 1.10)	1.03 (0.96, 1.11)	0.98 (0.91, 1.05)
Anxiolytic Medications	**1.05 (1.02, 1.09)**	1.03 (0.99, 1.08)	**1.06 (1.03, 1.10)**	**1.06 (1.02, 1.10)**	1.04 (1.00, 1.09)
Hypnotic Medications	1.04 (0.99, 1.08)	1.06 (0.98, 1.14)	1.03 (0.98, 1.08)	1.03 (0.98, 1.08)	**1.06 (1.00, 1.13)**
Mood-Stabilizer Medications	1.06 (1.00, 1.12)	1.07 (0.98, 1.16)	1.03 (0.94, 1.12)	**1.08 (1.00, 1.12)**	1.03 (0.94, 1.12)
Negative Control Outcome					
Statin Medications	1.02 (0.99, 1.05)	1.03 (0.97, 1.09)	1.02 (0.99, 1.05)	1.03 (0.99, 1.06)	1.03 (0.99, 1.06)

© 2024 Wettstein ZS et al. *JAMA Network Open.*

eFigure 1. Comparison of Mean Daily Change in Psychotropic Medication Prescriptions by different Pre-Fire and Fire Periods (Start Date Posted on Twitter), Across 25 Large California Wildfires in California MSAs: 2011-2018.

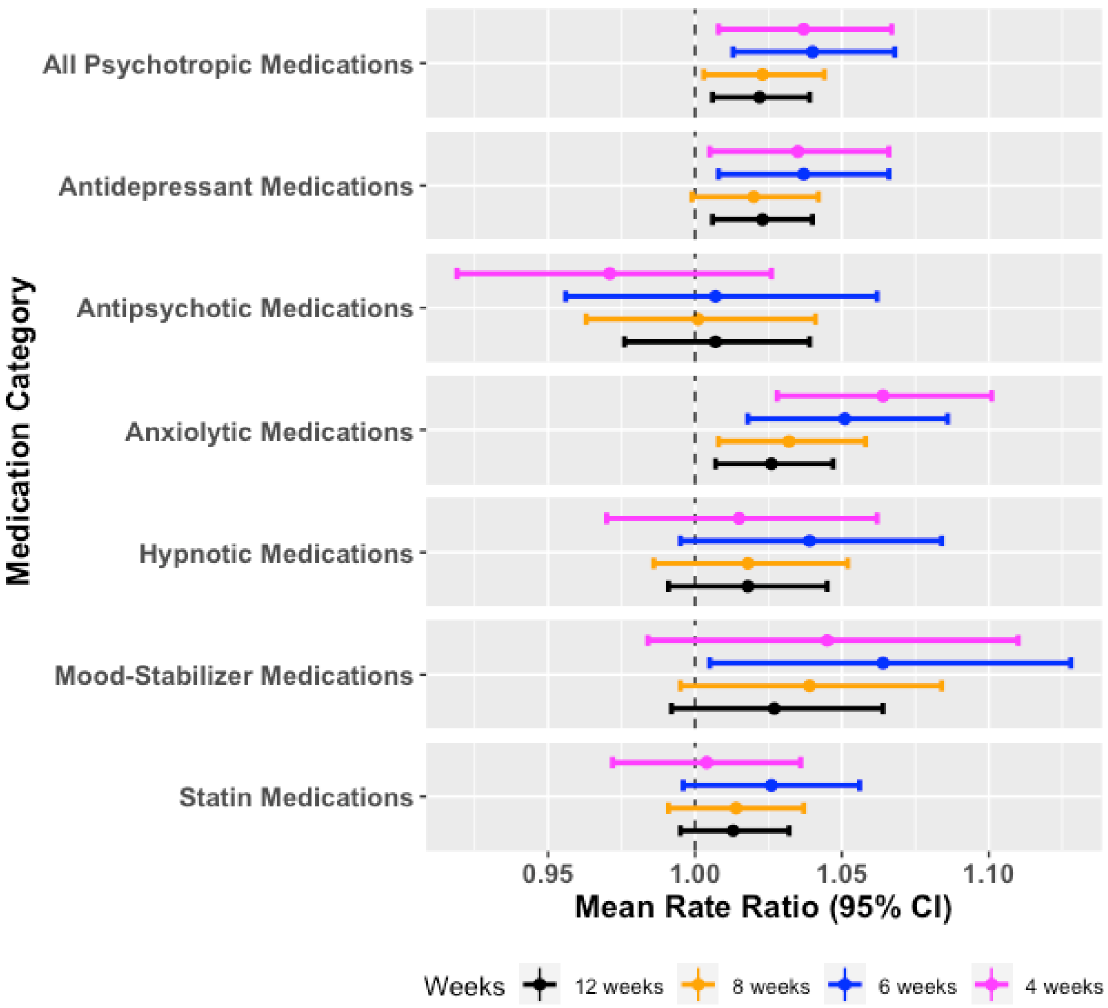

© 2024 Wettstein ZS et al. *JAMA Network Open.*

eFigure 2. Comparison of Mean Daily Change in Psychotropic Medication Prescriptions After Controlling for extreme weather events or disasters, meteorological variables, and air pollution parameters. (Start Date Posted on Twitter), Across 25 Large California Wildfires—Results from sensitivity analysis.

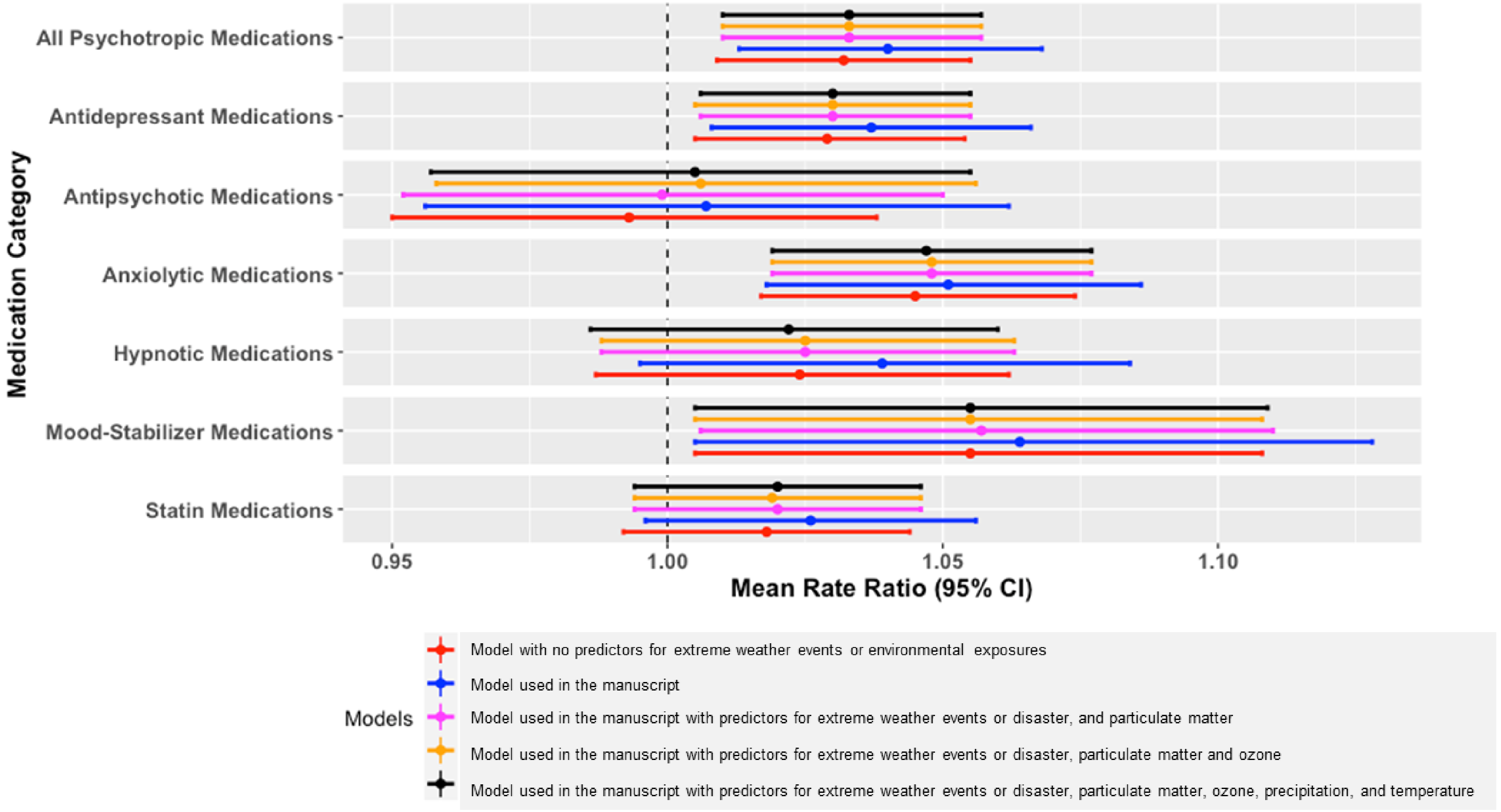

© 2024 Wettstein ZS et al. *JAMA Network Open.*
