# Supplementary material for: Psychotropic Medication Prescriptions and Large California Wildfires
Source: JAMA Netw Open. 2024 Feb 26;7(2):e2356466. doi: 10.1001/jamanetworkopen.2023.56466 (PMC10897744; doi:10.1001/jamanetworkopen.2023.56466)
Supplement: Supplement 2. — Data Sharing Statement [file jamanetwopen-e2356466-s002.pdf]

Data Sharing Statement

Wettstein. Psychotropic Medication Prescriptions and Large California Wildfires. *JAMA Netw Open*. Published February 22, 2024. doi:10.1001/jamanetworkopen.2023.56466

Data
Data available: No
